# Semantic Image Inpainting with Multi-Stage Feature Reasoning Generative Adversarial Network

**DOI:** 10.3390/s22082854

**Published:** 2022-04-08

**Authors:** Guangyao Li, Liangfu Li, Yingdan Pu, Nan Wang, Xi Zhang

**Affiliations:** School of Computer Science, Shaanxi Normal University, Xi’an 710062, China; lgy202649@snnu.edu.cn (G.L.); nhy@snnu.edu.cn (Y.P.); llmunan@snnu.edu.cn (N.W.); tshuajiang@snnu.edu.cn (X.Z.)

**Keywords:** deep learning, progressive image inpainting, hybrid weighted merging, point-wise normalization

## Abstract

Most existing image inpainting methods have achieved remarkable progress in small image defects. However, repairing large missing regions with insufficient context information is still an intractable problem. In this paper, a Multi-stage Feature Reasoning Generative Adversarial Network to gradually restore irregular holes is proposed. Specifically, dynamic partial convolution is used to adaptively adjust the restoration proportion during inpainting progress, which strengthens the correlation between valid and invalid pixels. In the decoding phase, the statistical natures of features in the masked areas differentiate from those of unmasked areas. To this end, a novel decoder is designed which not only dynamically assigns a scaling factor and bias on per feature point basis using point-wise normalization, but also utilizes skip connections to solve the problem of information loss between the codec network layers. Moreover, in order to eliminate gradient vanishing and increase the reasoning times, a hybrid weighted merging method consisting of a hard weight map and a soft weight map is proposed to ensemble the feature maps generated during the whole reconstruction process. Experiments on CelebA, Places2, and Paris StreetView show that the proposed model generates results with a PSNR improvement of 0.3 dB to 1.2 dB compared to other methods.

## 1. Introduction

Image inpainting aims to reconstruct the missing regions of damaged images and make the repaired image reasonable in both structure and texture. This technology shows a promising performance in many applications, such as image restoration, concealing errors and retouching photos [1,2,3]. Traditional inpainting methods [4,5,6,7] can usually synthesize relatively reasonable stationary textures. However, without semantic understanding of images, they are impossible to generate visually realistic content when the scenes are complex.

Recently, the methods based on encoder-decoder architecture [8,9,10,11,12,13] have massively improved the inpainting performance; the U-Net structure especially demonstrated a strong ability to generate detailed images. These methods encoded the input image into a latent high-level feature space, and then decoded it back to low-level pixels to fill the missing area in one shot. They are more suitable for filling images with a small range of holes, since the pixels within the local area have a strong correlation. However, as the damaged regions become larger, the model lacks effective features to infer missing contents, one-shot filling will generate semantically ambiguous results. An alternative solution is progressive inpainting. These methods divide the whole restoration process into several phases, each of which employs the information of previous phases as clues to restore the missing area step by step. For example, PGN [14] progressively inpaints the missing regions from the hole boundary to center, but the reconstruction at the image level suffers from high computational cost and information distortion. In order to reduce the amount of calculation and strengthen the connection between features, RFR-Net [15] supposes to share the attention scores of adjacent recurrences. However, there are still several limitations which could impact the performance of the existing progressive inpainting solutions, and they are summarized as follows. Firstly, current methods ignore the characteristic that the hole regions will gradually shrink. It means that the correlation between valid and invalid pixels will be weakened since the receptive field is fixed which is used to update the mask at different phases. Secondly, the mean and variance of pixels in the valid regions could be different from those of hole regions. Using batch normalization would result in covariant shift. Finally, progressive image inpainting gradually repairs the image by multiple recursions, so early generated signals will corrupt after long term propagation. Using adaptive average merging would degrade the quality of the generated features.

**Figure 1:**
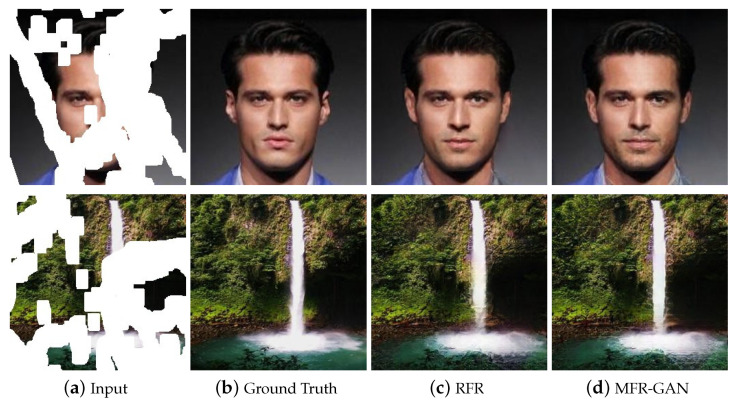
Comparing the proposed method with the state-of-the-art progressive image restoration method [15]. The missing regions are shown in white.

To address these issues, a novel image inpainting framework named Multi-stage Feature Reasoning Generative Adversarial Network (MFR-GAN) is proposed in this paper, which has the ability to generate more realistic and visually pleasing results, as shown in Figure 1. In view of the missing regions gradually narrowing down during the progressive inpainting process, we use the dynamic partial convolution which can regulate the restoration scale according to the scope of damaged areas. By this means, the correlation between known and unknown pixels is enhanced. In the process of progressive image inpainting, the input features will be decoded and encoded multiple times. Since the mean and variance of valid and invalid pixels are different, we designed a new decoding structure which not only leverages point wise normalization instead of batch normalization, but also uses skip-connection to minimize the loss of context information during decoding. To avoid covariant shift as mentioned above, point wise normalization is realized by adaptively assigning scale factors and biases to each feature point in the upsampling process.

When the reconstruction process is completed, the intermediate information would be ignored if directly using the last recurrence feature map as the final result. Moreover, pixels of the recovered regions are changed during the subsequent inpainting processes. This means that it is difficult to guarantee that the correct clues are always synthesized in intermediate restorations. If wrong information is generated at a certain step, it will be inherited and become worse at the following steps. To tackle these issues, we proposed a hybrid weighted merging method. It is constituted of a hard weight map and a soft weight map. The hard weight map is obtained by analyzing mask characteristics, which could enhance the influence of signals generated in the earlier reconstruction phases. Moreover, our model should be learnable and have the ability to pay attention to certain areas of a reconstructed feature map. To this end, the soft weight map is designed for achieving more realistic content. For the image discriminator, we use the Patch GAN [16] architecture which enables the model to pay more attention to image details during the training process. The main novelties and contributions of this work can be summarized as follows:

(1) We design a novel Multi-stage Feature Reasoning Generative Adversarial Network, which can adaptively adjust the inpainting scope in the recursive process through dynamic partial convolution, and leverage point wise Normalization to avoid covariant shift caused by the batch normalization.
(2) In the image fusion phase, we propose a hybrid weighted merging method that accurately merges the feature maps generated in each recurrence. By this means, we eliminated the problem of the gradient vanishing and the destruction of the content generated in the previous.
(3) Experiments on the benchmark datasets show that our MFR-GAN has effectively boosted the inpainting performance and generated semantically reasonable content.

The rest of the paper is organised as follows. Section 2 introduces the related work of image inpainting; Section 3 describes methods of the present study; Section 4 is concerned with the main results and the ablation study; Section 5 presents the conclusions.

## 2. Related Work

Traditional image inpainting methods are mainly composed of two categories: patch-based and diffusion-based. The patch-based inpainting methods [17] filled missing areas by calculating the similarity between patches and transferring similar areas from the background area to the hole area. The diffusion-based inpainting methods [18] attempted to propagate neighboring information to the corrupted areas. Due to capability limitation as well as as lack of semantic understanding of the image, these methods suffer from blurring artifacts when restoring relatively large regions.

Recently, deep learning based methods [19,20,21,22,23,24,25,26] have improved the capability of models to repair complex semantic environments. Context-Encoder [27] firstly employed the deep learning based method, which adopted an encoder-decoder based structure and used GAN [28] for image restoration. Shift-Net [29] introduced a special shift connection layer with the U-Net structure to fill arbitrary masked regions. PEN-Net [30] filled the holes from low resolution to high resolution with a U-shaped pyramid structure to boost the inpainting result. PConv [31] only used valid pixels to infer corrupted regions. GConv [12] further generalized partial convolution to gated convolution that learns to select features for feature maps at each level. Iizuka et al. [32] used a global and local discriminator for adversarial training to obtain coherent filling content. Liu et al. [33] designed region normalization to eliminate the influence of damaged regions on normalization. These methods cannot effectively settle the problems of semantic ambiguity, because they try to reconstruct the entire target without a strong correlation between the hole center pixels and the hole boundary pixels.

SPG-Net [34] factorized image restoration into segmentation prediction and guidance. EdgeConnect [9] utilized the hallucinated edge of the missing area for restoration to ensure structural consistency. Similarly, Xiong et al. [35] used contour and image completion to gradually recover the missing regions. StructureFlow [36] consisted of a texture and structure generator. The structure reconstructor removed high-frequency textures to restore the global structure, and then used appearance flow to synthesize image details. Li et al. [37] designed the visual structure reconstruction layer to restore part edges of a missing area for assisting the completion tasks. Yu et al. [13] devised the contextual attention and leveraged a coarse-to-fine framework to restore damaged images. These methods try to guide image inpainting by adding structural constraints, but they still lack adequate information to reconstruct the central area of the hole. Zhang et al. [14] leveraged U-Net generator with LSTM to concatenate all sub-tasks, and progressively filled the image with the corresponding output sequence. Guo et al. [38] used continuous full-resolution residual blocks to directly fill the missing area of the original size image. Li et al. [15] employed Knowledge Consistent Attention to adaptively combine the attention scores of different recurrent processes to improve the accuracy of image restoration. Although these methods have achieved considerable progressive, but they are still suffering from limitations described in the introduction.

## 3. Methods

**Figure 2:**
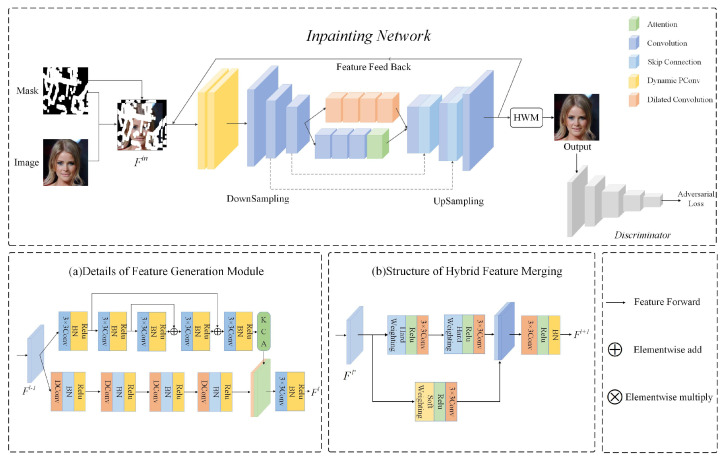
Illustration of our framework. Firstly, we use dynamic partial convolution to update the input mask $m^{l}$, then use the inpainting network to generate the pixels for the missing areas. Next, the above operations are performed repeatedly to generate the feature maps of different inpainting stages. Finally, the feature maps of each stage are fused by using the Hybrid weighted merging to generate the repaired results.

Figure 2 shows the overall architecture of the Multi-stage feature generation Adversarial network, whose inputs are the damaged image $X^{in}$ and the corresponding binary mask $M^{in}$, which indicates the missing regions. The proposed model is composed of three components: a feature generator to fill the holes in the feature maps, a feature merging model to accurately fuse the pixels synthesized in every recurrence, and a discriminator used for detail generation. We empirically use two parallel encoders to acquire semantic and image global structure information during the generation process. Firstly, we utilize the dynamic partial convolution to identify the region to be reconstructed in each recurrence. Next, the above operations are performed repeatedly to generate feature maps of different inpainting stages. When the corrupted images are completely filled, the feature maps of each stage are fused by the hybrid weighted merging model to generate the repaired results. Finally, the repaired image is sent to the discriminator for evaluating whether each patch belongs to the real or fake distribution, so as to improve the quality of image inpainting.

### 3.1. Hybrid Weighted Merging

**Figure 3:**
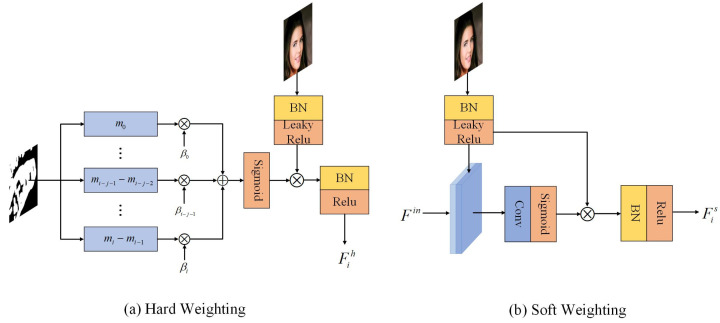
The details of the hybrid weighted merging method.

After a specific number of recurrences, the corrupted image has passed through the feature generation module several times. If we directly use the feature map generated by the last recurrence, it will cause the gradient to vanish and the loss of intermediate generated features. If adopting average merging, the defect area in early reconstructed image will damage subsequently generated information. The early-stage feature information is transmitted farther in the subsequent process to infer the center content; error information may be generated. Therefore, the signals generated in early stages at the same location should be more deterministic and the influence of signals generated in the later recurrences should be decreased. However, adaptively average fusing the valid pixels will affect the early generated feature information. To address this problem, we propose a hybrid weighted merging method to fuse the feature maps generated during hole restoration processes. It is composed of hard weighting and soft weighting, as shown in Figure 3. For hard weighting, on the premise of fusing non-hole region feature values, the model adaptively generates the weight proportion for current recurrence feature map according to the generation order of valid pixels. Firstly, divide the feature map as shown in the following formula:

(1) $$mask=\left\{\begin{array}{ll} mask_{i}, & \mathrm{if}\;j=0\\ mask_{i}-mask_{i-1}, & \mathrm{otherwise}, \end{array}\right.$$

where $mask_{i}$ is the mask of the $i^{th}$ recurrence update, $mask_{i-1}$ is the mask of the ${\left(i-1\right)}^{th}$ recurrence update. Then, a weight map $W_{i}$ is constructed for each recursively generated feature map ${F^{\prime }}^{i}$:

(2) $$W_{i}^{j}=\sum_{j=0}^{N}mask_{j}\times \frac{1}{1+e^{\left(S-i)/(S-j\right)}},$$

where *i* is the recurrence times, *j* is the number of regions, $mask_{j}$ is the $j^{th}$ region of $mask_{i}$. $W_{i}^{j}$ represents the $j^{th}$ region of the weight map corresponding to the $i^{th}$ recursive feature map ${F^{\prime }}^{i}$, and *S* is the number of feature maps generated in this inpainting process. Then, we use the softmax function to generate the proportion of the component $w_{x,y}^{i}$ for the pixel at position (x,y). $f_{x,y,z}$ represents the feature value of feature map ${F^{\prime }}^{i}$ at location (x,y,z), and the value of output feature map $\bar{F}$ at location (x,y,z) can be expressed as follows:

(3) $$f_{x,y,z}^{h}=\sum_{i=0}^{S}w_{x,y,z}^{h}\times \delta \left(\frac{f_{x,y,z}^{i}-\mu_{z}}{\sqrt{\sigma_{z}^{2}+\varepsilon }}\right).$$

**Figure 4:**
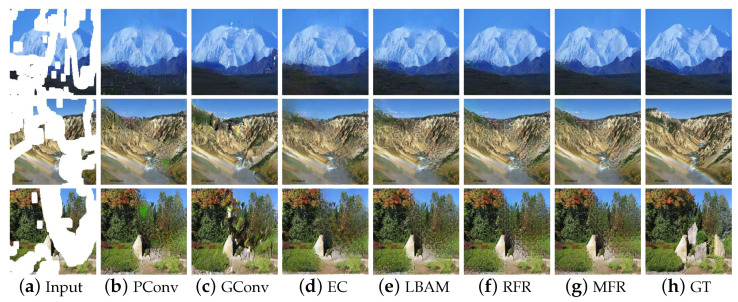
Qualitative comparison on Places2.

The hard weight map directly generates the weight by analyzing the mask without the learning process, which would limit the network’s performance. To this end, we propose a soft weight map to assist the hard weighting for achieving better inpainting results. The soft weight map is an adaptive map which is acquired by the input feature and average fused output feature map with a learning process. As shown in Figure 4, we concatenate the $F^{i}$ and input feature map $F^{in}$ to obtain a soft weight map:

(4) $$W^{s}=\sigma \left(\mathrm{Conv}\left(\left[F^{i},F^{in}\right]\right)\right)\cdot \left(1-M\right)+M,$$

where σ is the sigmoid activation function and the value of the feature map at location (x,y) after soft weighting can be expressed as follows:

(5) $$f_{x,y,z}^{s}=\sum_{i=0}^{S}w_{x,y,z}^{s}\times \delta \left(\frac{f_{x,y,z}^{i}-\mu_{z}}{\sqrt{\sigma_{z}^{2}+\varepsilon }}\right),$$

in which $\mu_{z}=\frac{1}{H\times W}\sum_{x=1}^{H}\sum_{y=1}^{W}f_{x,y,z}^{i}$, $\sigma_{z}=\sqrt{\frac{1}{H\times W}\sum_{x=1}^{H}\sum_{y=1}^{W}{\left(f_{x,y,z}^{i}-\mu_{z}\right)}^{2}}$, δ represents a leaky relu activation function. By fusing feature maps in the above manner, the gradient vanishing can be effectively avoided, improving the ability of MFR-GAN to restore large damaged areas.

### 3.2. Dynamic Partial Convolution

The significance of the restoration proportion is most noticeable when a model is applied to progressively inpaint the masked image. During the mask updating phase, in order to adaptively identify the area to be repaired in each recurrence, we introduce the dynamic partial convolution. It firstly calculates the holes ratio according to the input mask, and the formula is as follows:

(6) $$D=\frac{\sum_{i=0}^{N}E_{1,w}\Psi {}_{j}^{i}E_{h,1}}{\sum_{i=0}^{N}w\times h},$$

where *D* is the scale factor, $E_{1,w}$ and $E_{h,1}$ represent the 1×W row vector and H×1 column vector with the value of 1. $\Psi_{j}^{i}$ represents the mask corresponding to the input image $F^{in}$, *W* and *H* are the width and height of $\Psi_{j}^{i}$, and *N* represents the value of batch size. Next, the receptive field γ of convolution kernel is obtained by *D*, its formula is as follows:

(7) $$\gamma =\left\{\begin{array}{cc} \lceil D/1e^{-1}\rceil +2, & \;\mathrm{if}\;\beta \%2\neq 0\\ \lceil D/1e^{-1}\rceil +1, & \;\mathrm{if}\;\beta \%2=0 \end{array}\right.$$

After obtaining the value of γ, the next procedure is updating the area to be repaired. We set stride to 1 and padding = $\left(\gamma -1\right)/2$ ensures that the mask size is consistent with the feature map size, its formula is as follows:

(8) $$f_{x,y,z}=\left\{\begin{array}{cc} W^{T}\left(f_{x,y}⊙m_{x,y}\frac{sum(1)}{sum\left(m_{x,y}\right)}\right)+b, & \mathrm{if}\;\mathrm{sum}\left(m_{x,y}\right)>0\\ 0, & \mathrm{otherwise} \end{array}\right.$$

Here, $f_{x,y,z}$ is the feature value at location (x,y) of layer *z*; $W^{T}$ denotes the weight of the convolution layer filter, $f_{x,y}$ is the input feature patch of the current sliding window, $m_{x,y}$ is the input mask patch corresponding to $f_{x,y}$, 1 refers to a H×W matrix with all elements being 1, $\frac{sum(1)}{sum\left(m_{x,y}\right)}$ is the scale factor, the output result is adjusted when the number of convolution effective input values changes. The feature value of the new mask are expressed as:

(9) $$m_{x,y}^{*}=\left\{\begin{array}{c} 1,\;\mathrm{if}\;\mathrm{sum}\;\left(m_{x,y}\right)>0\\ 0,\;\mathrm{otherwise}\; \end{array}\right.$$

After dynamic partial convolution, the updated mask $M^{l}$ and the feature map $F^{l}$ are sent to the feature generation module. The difference between the updated mask and the input mask is defined as the area to be repaired in this iteration, and the updated mask remains unchanged until the next recurrence.

### 3.3. Feature Generation

A well-designed generator is vital to infer the missing content of the image. In order to fill the hole regions with high-quality features, two parallel encoders are used after down-sampling. The first encoder $E_{A}$ uses an attention mechanism to synthesize visually realistic textures, and the second encoder $E_{D}$ uses dilated convolution to collect the spatial features of the feature map. For encoder $E_{A}$, we use several convolution layers which are bridged through a skip connection, and apply knowledge Consistent Attention to control the inconsistencies between the adjacent attention feature maps. For encoder $E_{D}$, we directly stack four dilated convolution layers. After the input feature maps passing through $E_{A}$ and $E_{D}$, the outputs of the two encoders are concatenated and sent into a single decoder for up-sampling.

### 3.4. Attention Module

In image inpainting, the attention mechanism can search for possible textures in the background and use them to replace textures in unknown areas. It thus ensures that the filling contents are meaningful in both structure and texture. When the feature map $F^{i}$ is input into the attention model, we first calculate the cosine similarity between each pair of feature pixels:

(10) $${\hat{sim}}_{x,y,x^{\prime },y^{\prime }}^{i}=\langle \frac{f_{x,y}}{∥f_{x,y}∥},\frac{f_{x,y}}{∥f_{x,y}∥}\rangle ,$$

where ${\hat{sim}}_{x,y,x^{\prime },y^{\prime }}^{i}$ represents the similarity between features of the background image hole (x,y) and the foreground image hole $\left(x^{\prime },y^{\prime }\right)$. Then utilizing the similarity of the target pixels in the adjacent areas, we carry out k×k filtering to smooth the attention score:

(11) $$sim_{x,y,x^{\prime },y^{\prime }}^{{}^{\prime }i}=\frac{\sum_{p,q\in \{-k,\ldots ,k\}}{\hat{sim}}_{x+p,y+q,x^{\prime },y^{\prime }}}{k\times k}.$$

After that, the softmax function is used to generate the attention score, which is expressed as $score^{\prime }$. If the features at position (x,y) are valid in the last iteration, their attention scores are adaptively combined with present scores to synthesize the current iteration score:

(12) $$score_{x,y,x^{\prime },y^{\prime }}^{i}=score_{x,y,x^{\prime },y^{\prime }}^{{}^{\prime }i}.$$

If the pixel value at the (x,y) is invalid in the previous recurrence, the attention score obtained in this recursion is the final score:

(13) $$score_{x,y,x^{\prime },y^{\prime }}^{i}=\lambda score_{x,y,x^{\prime },y^{\prime }}^{i^{\prime }}+\left(1-\lambda \right)score_{x,y,x^{\prime },y^{\prime }}^{i-1}.$$

Next, the attention score is used to reconstruct the feature map $\hat{F}$. The feature value of $\hat{F}$ at (x,y) is ${\hat{f}}_{x,y}$, and the calculation process of ${\hat{f}}_{x,y}$ is as follows:

(14) $${\hat{f}}_{x,y}^{i}=\sum_{x^{\prime }\in 1,\ldots ,W^{\prime }\in 1,\ldots ,H}score_{x,y,x^{\prime },y^{\prime }}^{i}f_{x^{\prime },y^{\prime }}^{i}.$$

Finally, after splicing feature map $\hat{F}$ and input feature map $F^{in}$, a pixel-by-pixel convolution is performed to generate the reconstructed feature map $F^{\prime }$.

### 3.5. Point Wise Normalization

After the recovery decoder, the generated feature maps are sent for up-sampling. We infer that the statistical characteristics of pixels in the hole regions and the pixels in the no-hole regions are different. Using traditional batch normalization could ignore this characteristic and cause the covariant shift. To tackle this issue, we utilize point wise normalization in the decoding phase to dynamically produce the mask aware scale and bias of batch normalization. The input feature is first normalized in the channel wise manner, and then modulated with learned scale and bias.

(15) $${\bar{f}}_{x,y,z}^{i}=\gamma_{x,y,z}^{i}\left(m^{i}\right)\frac{f_{x,y,z}^{i}-\mu_{z}^{i}}{\sigma_{z}^{i}}+\beta_{x,y,z}^{i}\left(m^{i}\right),$$

where $f_{x,y,z}$ is the feature before normalization, $\mu_{z}$ and $\sigma_{z}$ are the mean and standard deviation of the activation in channel *z*.

### 3.6. Loss Function and Model Architecture

The entire training procedure is illustrated in Algorithm  1. We use a Patch-GAN [16] discriminator for image restoration learning. The Patch-GAN discriminator calculates the adversarial loss from the generator. The loss function is consisted of L1 loss, perceptual loss, style loss, adversarial loss and TV loss. L1 loss ensures the accuracy of feature map reconstruction. Given the binary mask with zeros indicating missing pixels, we define the L1 loss as follows:

(16) $$L_{hole}=\frac{1}{N_{I_{gt}}}{∥\left(1-M\right)⊙\left(I_{out}-I_{gt}\right)∥}_{1}$$

(17) $$L_{valid}=\frac{1}{N_{I_{gt}}}{∥M⊙\left(I_{out}-I_{gt}\right)∥}_{1},$$

where $I_{gt}$ and $I_{out}$ is the ground-truth image and output value of the network. $N_{I_{gt}}$ is the total number of elements in the image, which equals C×H×W.

The perceptual loss proposed by Gatys et al. [39] is used to force the filled image and the ground-truth image have similar feature representation. It can be written as follows:

(18) $$L_{perc}=\sum_{n=0}^{N-1}\frac{{∥\Psi_{n}^{I_{out}}-\Psi_{n}^{I_{gt}}∥}_{1}}{N_{\Psi_{n}^{I_{gt}}}}+\sum_{n=0}^{N-1}\frac{{∥\Psi_{n}^{I_{com}}-\Psi_{n}^{I_{gt}}∥}_{1}}{N_{\Psi_{n}^{I_{gt}}}},$$

where $\Psi_{n}$ represents the $n^{th}$ feature layer select in the fixed VGG, $I_{com}$ is composed of the hole range pixels of raw output image and non-hole pixels of the ground truth image. $N_{\Psi_{n}^{I_{gt}}}$ is the number of elements in $\Psi_{n}^{I_{gt}}$ and is used as a normalization factor.

A VGG-based [40] Style loss is similar to perceptual loss. The autocorrelation of each feature map is calculated before measuring the L1 distance, the computation of the style loss is as follows:

(19) $$L_{style_{out}}=\sum_{n=0}^{N-1}\frac{1}{C_{n}C_{n}}{∥K_{n}\left({\left(\Psi_{n}^{I_{out}}\right)}^{T}\left(\Psi_{n}^{I_{out}}\right)-{\left(\Psi_{n}^{I_{gt}}\right)}^{T}\left(\Psi_{n}^{I_{gt}}\right)\right)∥}_{1}$$

(20) $$L_{style_{com}}=\sum_{n=0}^{N-1}\frac{1}{C_{n}C_{n}}{∥K_{n}\left({\left(\Psi_{n}^{I_{com}}\right)}^{T}\left(\Psi_{n}^{I_{com}}\right)-{\left(\Psi_{n}^{I_{gt}}\right)}^{T}\left(\Psi_{n}^{I_{gt}}\right)\right)∥}_{1}.$$

**Algorithm 1:** Multi-stage Feature Reasoning GAN
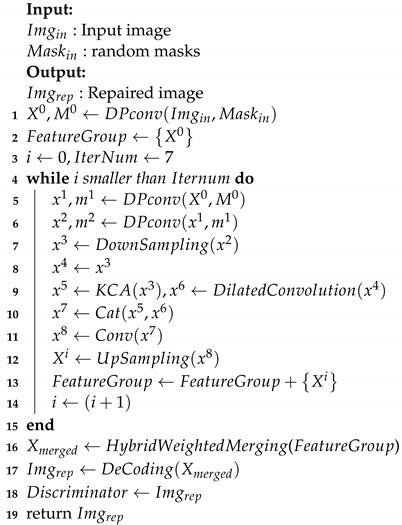


In (19) and (20), $K_{n}$ is the normalization factor for the $p^{th}$, equal to $1/C_{n}H_{n}W_{n}$. The final loss term total variation loss is expressed as follows:

(21) $$L_{tv}=\sum_{(i,j)\in R,(i,j+1)\in R}\frac{1}{N_{I_{com}}}∥I_{com}^{i,j+1}-I_{com}^{i,j}∥+\sum_{(i,j)\in R,(i+1,j)\in R}\frac{1}{N_{I_{com}}}∥I_{com}^{i+1,j}-I_{com}^{i,j}∥,$$

where $N_{I_{com}}$ represents the number of pixels in $I_{com}$. Total loss $L_{total}$ is the combination of the above loss functions:

(22) $$L_{total}=\lambda_{valid}L_{valid}+\lambda_{hole}L_{hole}+\lambda_{perc}L_{perc}+\lambda_{style}L_{style}+\lambda_{adv}L_{adv}+\lambda_{tv}L_{tv}.$$

In this paper, the weight parameters of each loss function in Equation (22) are set as follows: 1 for $\lambda_{valid}$, 6 for $\lambda_{hole}$, 0.1 for $\lambda_{tv}$, 120 for $\lambda_{style}$, 0.1 for $\lambda_{adv}$ and 0.05 for $\lambda_{perc}$.

## 4. Experiments

This section starts with the introduction of detailed experimental settings, then we compare our model with other methods in terms of both visual quality and quantitative measurements to demonstrate the efficiency of our proposed method. Finally, we conduct an ablation study to examine the design details of MFR-GAN.

### 4.1. Datasets

We used three well-known public image datasets and a mask dataset [31] to verify our model. Images were resized to 256×256 for training and testing.

• Places2 Dataset [41]: a dataset containing over 365 scenes, which enables the model to learn the distribution from many natural scenes. Including 1.8 million images for training and 12K images for testing.
• CelebA Dataset [42]: a dataset that focuses on face images, containing about 200K images. We used more than 180K images training models, and the other images were used for testing.
• Paris StreetView [43]: a dataset collected from Street View in Paris that is commonly used for inpainting methods. It contains 14,900 images for training and 100 images for testing.

### 4.2. Training Settings

For the MFR-GAN, we used the Adam optimizer to optimize the generator and discriminator. We trained the model with a batch size of 6 on 2 11G NVIDIA RTX2080Ti GPUs. At the beginning, we trained the model with a learning rate of $1\times 10^{-4}$. Then we set the learning rate to $1\times 10^{-5}$ for fine tuning the model, and it was kept unchanged until the model convergence. For CelebA, we first trained 200 K times, then fine tuned 500 K times until convergence. Pytorch was used as the deep learning development framework, and the CUDA version was v10.0.

### 4.3. Comparison Method

We compared our model with several recent state-of-the-art methods. These models are: PConv [31], GatedConv [12], EdgeConnect [9], LBAM [44] and RFR [15]. For LBAM, EdgeConnect, RFR-Net and GConv, we directly used the officially released pretrained model. Since the source code of partial convolution was not available, we implemented it with the experimental settings in the paper. Compared with PConv, GConv, LBAM and EC, the proposed method strengthens the constraint on the center of the hole by progressively reasoning the content of the hole from the edge of the hole. Compared with RFR-Net, the proposed method solves the problem of covariant shift by using point-wise normalization, and a hybrid fusion method is introduced to make full use of the feature maps generated at each stage, which effectively improves the ability of the model to repair large holes.

### 4.4. Qualitative Comparisons

For qualitative comparisons, we compared the irregular holes inpainting results of our method with five existing methods on the Places2, CelebA and Pairs StreetView datasets. Figure 4 shows that there are varying degrees of blurry boundary and distorted structures when PConv and Edgeconnect repair natural images. Moreover, when PConv repairs the semantic complex natural image, the color distortion is relatively serious, and neither structural connectivity of the generated content nor smooth and reasonable color content can be achieved. GConv well preserves the source image contents, but there are still color inconsistencies in some areas of the result. The result of LBAM suffers from center blur due to the lack of information for restoring deeper pixels in holes. Although RFR-Net can generate meaningful content through leveraging the learned intermediate signals for further restoration, the results still have unreasonable textures. In contrast, the results generated by our model have reasonable semantics and visual authenticity.

**Figure 5:**
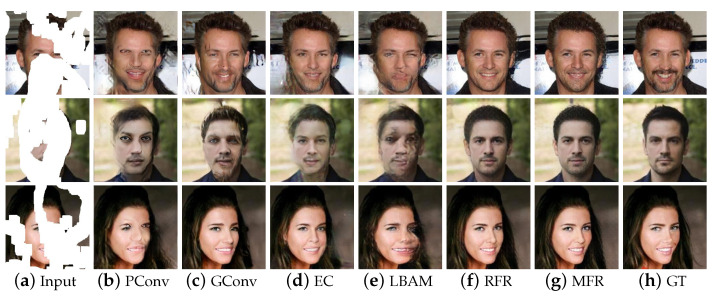
Qualitative comparison of CelebA.

Figure 5 shows the inpainting results on CelebA; it is observed that content synthesized by PConv, LBAM and GConv is relatively vague, and the color of some areas is different from the original image. When the holes are relatively large, the image edge generated in the first stage of EdgeConnect includes error information, which leads to the failure of generating a correct structure in the second stage. RFR-Net can generate a plausible structure, but the results still contain unreasonable textures. Through the flexible combination of point wise normalization and skip-connection during the decoding process, our model could make full use of contextual feature information, and generates the content with reasonable semantics and rich details.

**Figure 6:**
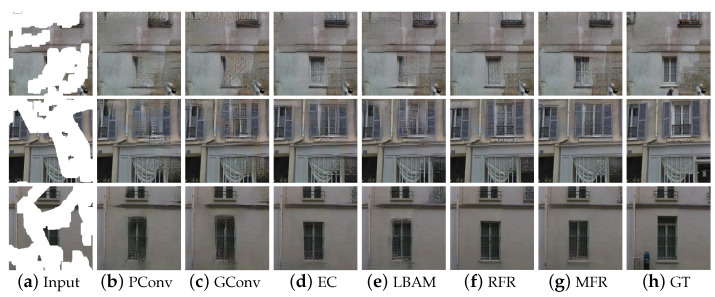
Qualitative comparison on Paris StreetView.

The Paris StreetView Dataset contains images of highly complex structures. As shown in Figure 6, when too much valid information is missing, there are obvious artifacts in the inpainting results of PConv and GConv. LBAM, EC and RFR can generate natural structures, but the results still have unsmooth content. Compared with the above methods, the texture of the inpainted regions is more natural in our results. These results suggest that our method can learn to synthesize better signals by making full use of mask information to gradually fill the missing contents. Besides, in the feature fusion phase, on the basis of eliminating gradient vanishing, the hybrid weighted merging method enables the model to adaptively fuse the feature maps with a learning process, which greatly enhances the reasoning capability of the model.

### 4.5. Quantitative Comparisons

**Figure 7:**
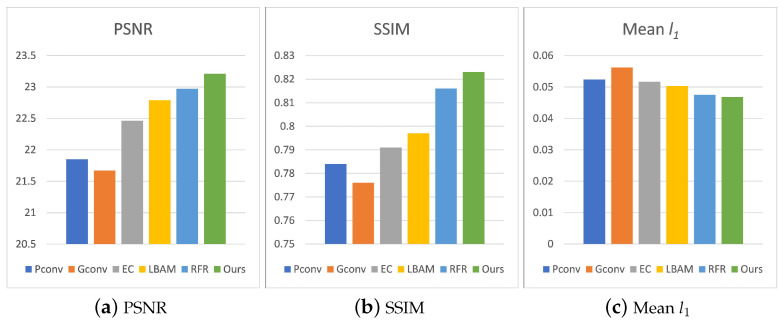
Quantitative comparison of CelebA, the mask ratio is between 0.5 and 0.6.

Compared with other datasets, Places2 contains more scenarios, thus it can better verify the authenticity of different methods. For the evaluation metrics, we use peak signal-to-noise ratio (PSNR) to measure the L2 distance. The larger the value of PSNR, the better the image restoration effect is. Usually, when the PSNR value is greater than 28, there is no significant difference in image quality. SSIM is used to measure structural similarity; its value range is between 0 and 1. The higher value means less image distortion. Fréchet Inception Distance (FID) is used to measure the Wasserstein-2 distance between fake and real images. A lower FID score indicates that the two sets of images are more similar, and a score of zero in the best case indicates that the two sets of images are the same. We use the same irregular mask as PConv for testing. The masks are divided into six categories according to the proportion of holes: (0.01,0.1], (0.1,0.2], (0.2,0.3], (0.3,0.4], (0.4,0.5], (0.5,0.6]. For each category, we randomly use 50 masks and images as input to test the model. For the performance of repairing irregular masks, as shown in Table 1, when the missing ratio is (0.2,0.3], the average PSNR of the repair results is 26.23 dB, the SSIM is 0.922, and the FID is 12.79. Our method has produced excellent results. When the hole ratio is (0.5,0.6], the PSNR is 19.53 dB, the SSIM is 0.669, and the FID is 34.27. Although the effective information is insufficient, our model can still generate clear content through multiple inferences, as shown in Figure 7 and Table 1. This further validates the effectiveness of our method.

### 4.6. Ablation Study

In this section, we conduct ablation experiments on the Places2 datasets, and illustrate the effectiveness of dynamic partial convolution, hybrid weighted merging, point wise normalization and the effect of recurrence number based on PSNR, SSIM, and FID.

**Figure 8:**
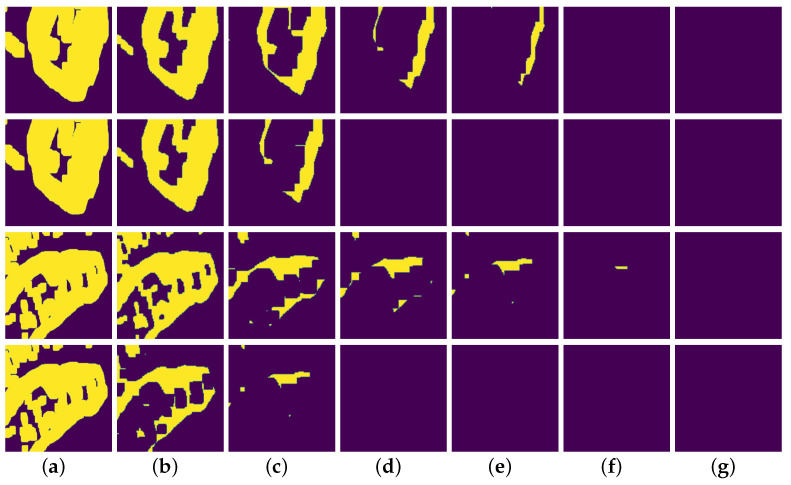
Visualization of the mask updated during the inpainting process. Yellow areas represent invalid pixels, and purple areas denote valid pixels. From the left to the right are: (**a**) Input mask, (**b**) first updated mask, (**c**) second updated mask, (**d**) third updated mask, (**e**) fourth updated mask, (**f**) fifth updated mask, (**g**) sixth updated mask.

**Dynamic partial convolution** As demonstrated in Figure 8, we visualize the changes of mask during the whole restoration process. The first row and third row show the mask variation of our model during the inpainting process, and the second row and fourth row are RFR-Net. It is shown that the hole change in our method is smoother than that of RFR-Net. Specifically, our model has learned to dynamically adjust the size of receptive field according to the mask ratios for updating the mask, which is beneficial for strengthening the connection of corrupted area pixels and valid pixels during the progressive completion process.

**Figure 9:**
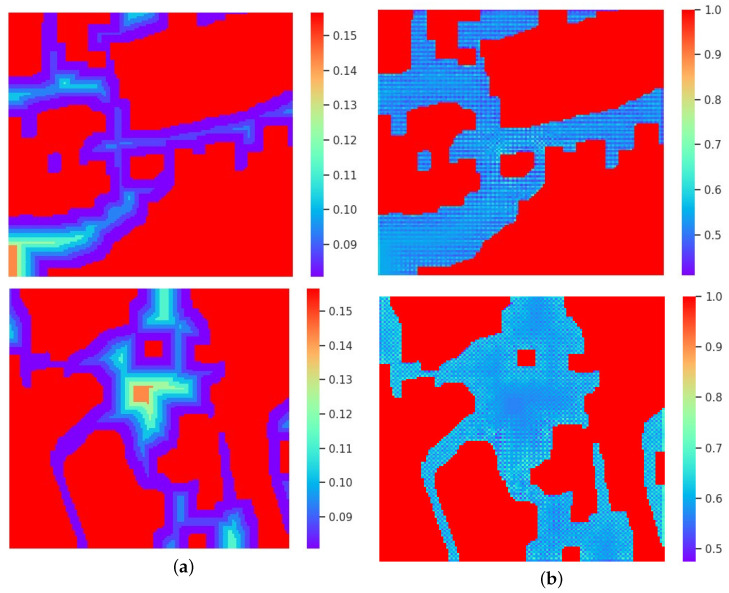
The visualization of the hard weight map and soft weight map. (**a**) is the hard weight map and (**b**) is the soft weight map.

**Hybrid weighted merging** The hybrid weighted merging method effectively increases the reasoning times. For large holes, the model can perform multiple inferences for generating more realistic content. Figure 9 shows the visualization of the hard weight map and the soft weight map. The weights of the hard weight map are obtained by the generated order of the signals, as shown in Figure 9a; because the center is repaired in the later recurrence, thus the weight gradually increases from the hole boundary to the center. As shown in Figure 9b, the weight distribution of the soft weight map is uniform, and the weights are adaptively changed by borrowing information from the input features. BN+G AW is a U-Net architecture using average merging, which achieves a PSNR of 21.83 dB. We replace the average merging with hybrid weighted merging, which is named BN+G HW. As shown in Table 2, under the same training strategies and experimental settings, BN+G HW achieves an average PSNR of 21.89 dB, SSIM of 0.808 and FID of 23.82. We can see that the quantitative score is significantly improved after using the hybrid weighted merging method.

**Figure 10:**
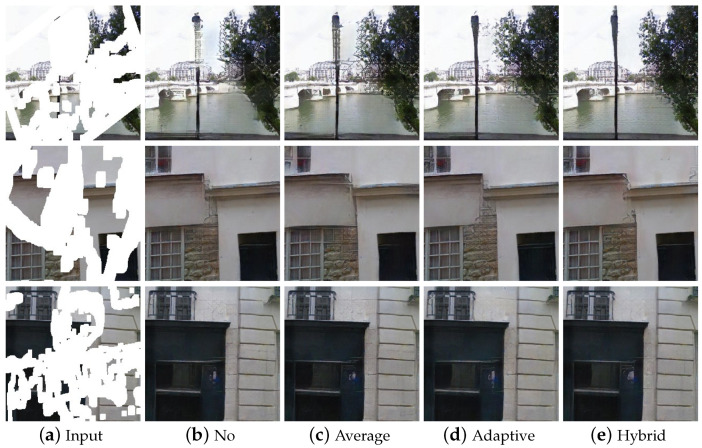
Different methods for feature merging. From the left to the right are: (**a**) Input, (**b**) No merging, (**c**) Average merging, (**d**) Adaptive merging, (**e**) Hybrid merging.

As shown in Figure 10, we compared the hybrid weighted merging method with other feature fusion methods on the Paris StreetView dataset, which has more repetitive structures such as gates. It can be observed that the image structure generated by our method has clear textures and consistent contextual structures.

**Point wise normalization.** The results in the third row and fourth row demonstrate the performance with point wise normalization. Compared to the Batch Normalization counterpart, the PSNR and SSIM are significantly improved, this means that point-wise normalization is able to capture the discrepancy between valid and invalid pixels during decoding processes, and learn the adaptive scale and bias parameters for assisting batch normalization.

**The effect of iteration numbers** As a hyper-parameter, the IterNums is set to seven in our model. In order to demonstrate the influence of different IterNums, we conduct the experiments on the Places2 dataset with large continuous holes. As shown in Table 3, the results from IterNums 5 are far from the others. This is because it is difficult to completely restore the large continuous holes in the small IterNums. Too many recursion times, resulting in far feature propagation, is also not conducive to image restoration.

## 5. Conclusions

In this paper, we propose a novel Multi-stage Feature Reasoning Generative Adversarial Network using recurrence filling to restore the arbitrary image defects. The hybrid weighted merging method fuses the feature map base to the mask characteristic and a learning process; it takes full advantage of the signals generated in every recurrence and thus eliminates gradient vanishing and increases the reasoning times of the model. Through the dynamic partial convolution, the image restoration range is adaptively adjusted according to the mask ratio. By this means, the correlation between hole boundary pixels and center area pixels is gradually strengthened, which is especially suitable for progressive image completion. Furthermore, skip-connection and point wise normalization are combined to minimize the loss of valid information in the up-sampling process; thus, the generated result structure is clearer and the content is more natural. Extensive experiments on the Places2, CelebA and Pairs StreetView datasets have demonstrated that MFR-GAN is more competitive than other methods in subjective quality and is objectively quantitative.

## Figures and Tables

**Table 1:** Numerical comparisons on the Place2 dataset. ↑ indicates higher is better while ↓ indicates lower is better.

	Methods	(0.01, 0.1]	(0.1, 0.2]	(0.2, 0.3]	(0.3, 0.4]	(0.4, 0.5]	(0.5, 0.6]
	PConv	34.15	27.68	25.23	22.34	21.35	18.71
	GC	34.03	27.12	24.95	22.13	20.92	18.35
PSNR↑	LBAM	34.62	27.87	25.42	22.91	21.75	18.81
	EC	34.36	27.46	25.58	22.83	21.58	18.97
	RFR-Net	34.94	28.05	25.99	23.56	21.78	19.36
	Ours	35.31	28.29	26.23	23.83	21.95	19.53
	PConv	0.982	0.943	0.901	0.839	0.787	0.632
	GC	0.981	0.939	0.898	0.835	0.779	0.618
SSIM↑	LBAM	0.984	0.947	0.905	0.846	0.783	0.634
	EC	0.983	0.942	0.908	0.848	0.789	0.636
	RFR-Net	0.985	0.948	0.915	0.871	0.805	0.663
	Ours	0.987	0.954	0.922	0.877	0.811	0.669
	PConv	4.56	11.21	16.63	23.15	34.27	47.59
	GC	4.24	9.79	15.74	24.92	33.94	45.13
FID↓	LBAM	4.12	9.35	14.85	22.41	28.54	42.31
	EC	3.97	8.27	14.59	19.57	26.94	39.15
	RFR-Net	3.28	7.03	13.45	18.34	24.38	35.82
	Ours	2.93	6.81	12.79	16.85	23.26	34.27

**Table 2:** Quantitative ablation study on the Places2 dataset.

Method	PSNR	SSIM	FID
BN+G AW	21.83	0.806	24.21
BN+G HW	21.89	0.808	23.82
PN+G AW	21.92	0.809	23.54
PN+G HW	21.95	0.811	23.26

**Table 3:** The influences of different IterNums.

IterNums	PSNR	SSIM	FID
5	18.39	0.605	40.21
7	19.53	0.669	34.27
9	19.32	0.661	35.93

## Data Availability

Data sharing not applicable.

## References

[1] Shetty R.R., Fritz M., Schiele B. (2018). Adversarial scene editing: Automatic object removal from weak supervision. Adv. Neural Inf. Process. Syst..

[2] Song L., Cao J., Song L., Hu Y., He R. Geometry-aware face completion and editing. Proceedings of the AAAI Conference on Artificial Intelligence.

[3] Armi L., Fekri-Ershad S. (2019). Texture image analysis and texture classification methods—A review. arXiv.

[4] Barnes C., Shechtman E., Finkelstein A., Goldman D.B. (2009). PatchMatch: A randomized correspondence algorithm for structural image editing. ACM Trans. Graph..

[5] Ding D., Ram S., Rodríguez J.J. (2018). Image inpainting using nonlocal texture matching and nonlinear filtering. IEEE Trans. Image Process..

[6] Lee J.H., Choi I., Kim M.H. Laplacian patch-based image synthesis. Proceedings of the IEEE Conference on Computer Vision and Pattern Recognition.

[7] Criminisi A., Pérez P., Toyama K. (2004). Region filling and object removal by exemplar-based image inpainting. IEEE Trans. Image Process..

[8] Sagong M.c., Shin Y.g., Kim S.w., Park S., Ko S.j. Pepsi: Fast image inpainting with parallel decoding network. Proceedings of the IEEE/CVF Conference on Computer Vision and Pattern Recognition.

[9] Nazeri K., Ng E., Joseph T., Qureshi F., Ebrahimi M. Edgeconnect: Structure guided image inpainting using edge prediction. Proceedings of the IEEE/CVF International Conference on Computer Vision Workshops.

[10] Li C., He K., Liu K., Ma X. (2020). Image Inpainting Using Two-Stage Loss Function and Global and Local Markovian Discriminators. Sensors.

[11] Yeh R.A., Chen C., Yian Lim T., Schwing A.G., Hasegawa-Johnson M., Do M.N. Semantic image inpainting with deep generative models. Proceedings of the IEEE Conference on Computer Vision and Pattern Recognition.

[12] Yu J., Lin Z., Yang J., Shen X., Lu X., Huang T.S. Free-form image inpainting with gated convolution. Proceedings of the IEEE/CVF International Conference on Computer Vision.

[13] Yu J., Lin Z., Yang J., Shen X., Lu X., Huang T.S. Generative image inpainting with contextual attention. Proceedings of the IEEE Conference on Computer Vision and Pattern Recognition.

[14] Zhang H., Hu Z., Luo C., Zuo W., Wang M. Semantic image inpainting with progressive generative networks. Proceedings of the 26th ACM international conference on Multimedia.

[15] Li J., Wang N., Zhang L., Du B., Tao D. Recurrent feature reasoning for image inpainting. Proceedings of the IEEE/CVF Conference on Computer Vision and Pattern Recognition.

[16] Isola P., Zhu J.Y., Zhou T., Efros A.A. Image-to-image translation with conditional adversarial networks. Proceedings of the IEEE Conference on Computer Vision and Pattern Recognition.

[17] Darabi S., Shechtman E., Barnes C., Goldman D.B., Sen P. (2012). Image melding: Combining inconsistent images using patch-based synthesis. ACM Trans. Graph..

[18] Hays J., Efros A.A. (2007). Scene completion using millions of photographs. ACM Trans. Graph..

[19] Liu H., Jiang B., Xiao Y., Yang C. Coherent semantic attention for image inpainting. Proceedings of the IEEE/CVF International Conference on Computer Vision.

[20] Yi Z., Tang Q., Azizi S., Jang D., Xu Z. Contextual residual aggregation for ultra high-resolution image inpainting. Proceedings of the IEEE/CVF Conference on Computer Vision and Pattern Recognition.

[21] He X., Yin Y. (2021). Non-Local and Multi-Scale Mechanisms for Image Inpainting. Sensors.

[22] Dong H., Liang X., Zhang Y., Zhang X., Shen X., Xie Z., Wu B., Yin J. Fashion editing with adversarial parsing learning. Proceedings of the IEEE/CVF Conference on Computer Vision and Pattern Recognition.

[23] Liu H., Wan Z., Huang W., Song Y., Han X., Liao J. PD-GAN: Probabilistic Diverse GAN for Image Inpainting. Proceedings of the IEEE/CVF Conference on Computer Vision and Pattern Recognition.

[24] Suvorov R., Logacheva E., Mashikhin A., Remizova A., Ashukha A., Silvestrov A., Kong N., Goka H., Park K., Lempitsky V. Resolution-robust Large Mask Inpainting with Fourier Convolutions. Proceedings of the IEEE/CVF Winter Conference on Applications of Computer Vision.

[25] Zhu M., He D., Li X., Li C., Li F., Liu X., Ding E., Zhang Z. (2021). Image Inpainting by End-to-End Cascaded Refinement with Mask Awareness. IEEE Trans. Image Process..

[26] Yang S., Huang R., Han F. (2021). Progressively Inpainting Images Based on a Forked-Then-Fused Decoder Network. Sensors.

[27] Pathak D., Krahenbuhl P., Donahue J., Darrell T., Efros A.A. Context encoders: Feature learning by inpainting. Proceedings of the IEEE Conference on Computer Vision and Pattern Recognition.

[28] Zhao L., Mo Q., Lin S., Wang Z., Zuo Z., Chen H., Xing W., Lu D. Uctgan: Diverse image inpainting based on unsupervised cross-space translation. Proceedings of the IEEE/CVF Conference on Computer Vision and Pattern Recognition.

[29] Yan Z., Li X., Li M., Zuo W., Shan S. Shift-net: Image inpainting via deep feature rearrangement. Proceedings of the European Conference on Computer Vision (ECCV).

[30] Zeng Y., Fu J., Chao H., Guo B. Learning pyramid-context encoder network for high-quality image inpainting. Proceedings of the IEEE/CVF Conference on Computer Vision and Pattern Recognition.

[31] Liu G., Reda F.A., Shih K.J., Wang T.C., Tao A., Catanzaro B. Image inpainting for irregular holes using partial convolutions. Proceedings of the European Conference on Computer Vision (ECCV).

[32] Iizuka S., Simo-Serra E., Ishikawa H. (2017). Globally and locally consistent image completion. ACM Trans. Graph..

[33] Yu T., Guo Z., Jin X., Wu S., Chen Z., Li W., Zhang Z., Liu S. Region normalization for image inpainting. Proceedings of the AAAI Conference on Artificial Intelligence.

[34] Song Y., Yang C., Shen Y., Wang P., Huang Q., Kuo C.C.J. (2018). Spg-net: Segmentation prediction and guidance network for image inpainting. arXiv.

[35] Xiong W., Yu J., Lin Z., Yang J., Lu X., Barnes C., Luo J. Foreground-aware image inpainting. Proceedings of the IEEE/CVF Conference on Computer Vision and Pattern Recognition.

[36] Ren Y., Yu X., Zhang R., Li T.H., Liu S., Li G. Structureflow: Image inpainting via structure-aware appearance flow. Proceedings of the IEEE/CVF International Conference on Computer Vision.

[37] Li J., He F., Zhang L., Du B., Tao D. Progressive reconstruction of visual structure for image inpainting. Proceedings of the IEEE/CVF International Conference on Computer Vision.

[38] Guo Z., Chen Z., Yu T., Chen J., Liu S. Progressive image inpainting with full-resolution residual network. Proceedings of the 27th Acm International Conference on Multimedia.

[39] Gatys L.A., Ecker A.S., Bethge M. (2015). A neural algorithm of artistic style. arXiv.

[40] Simonyan K., Zisserman A. (2014). Very deep convolutional networks for large-scale image recognition. arXiv.

[41] Zhou B., Lapedriza A., Khosla A., Oliva A., Torralba A. (2017). Places: A 10 million image database for scene recognition. IEEE Trans. Pattern Anal. Mach. Intell..

[42] Liu Z., Luo P., Wang X., Tang X. Deep learning face attributes in the wild. Proceedings of the IEEE International Conference on Computer Vision.

[43] Doersch C., Singh S., Gupta A., Sivic J., Efros A. (2012). What makes paris look like paris?. ACM Trans. Graph..

[44] Xie C., Liu S., Li C., Cheng M.M., Zuo W., Liu X., Wen S., Ding E. Image inpainting with learnable bidirectional attention maps. Proceedings of the IEEE/CVF International Conference on Computer Vision.

